# Development of a Non-Destructive Method for Detection of the Juiciness of Pear via VIS/NIR Spectroscopy Combined with Chemometric Methods

**DOI:** 10.3390/foods9121778

**Published:** 2020-11-30

**Authors:** Fan Wang, Chunjiang Zhao, Guijun Yang

**Affiliations:** 1 Beijing Research Center for Information Technology in Agriculture, Beijing Academy of Agriculture and Forestry Sciences, Beijing 100097, China; wangf@nercita.org.cn (F.W.); yanggj@nercita.org.cn (G.Y.); 2 National Engineering Research Center for Information Technology in Agriculture, Beijing 100097, China; 3 Key Laboratory of Quantitative Remote Sensing in Agriculture of Ministry of Agriculture, Beijing 100097, China

**Keywords:** pear, juiciness, VIS/NIR spectroscopy, preprocessing

## Abstract

Juiciness is a primary index of pear quality and freshness, which is also considered as important as sweetness for the consumers. Development of a non-destructive detection method for pear juiciness is meaningful for producers and sellers. In this study, visible−near-infrared (VIS/NIR) spectroscopy combined with different spectral preprocessing methods, including normalization (NOR), first derivative (FD), detrend (DET), standard normal variate (SNV), multiplicative scatter correction (MSC), probabilistic quotient normalization (PQN), modified optical path length estimation and correction (OPLECm), linear regression correction combined with spectral ratio (LRC-SR) and orthogonal spatial projection combined with spectral ratio (OPS-SR), was used for comparison in detection of pear juiciness. Partial least squares (PLS) regression was used to establish the calibration models between the preprocessing spectra (650–1100 nm) and juiciness measured by the texture analyzer. In addition, competitive adaptive reweighted sampling (CARS) was used to identify the characteristic wavelengths and simplify the PLS models. All obtained models were evaluated via Monte Carlo cross-validation (MCCV) and external validation. The PLS model established by 19 characteristic variables after LRC-SR preprocessing displayed the best prediction performance with external verification determination coefficient (*R*^2^_v_) of 0.93 and root mean square error (*RMSE*_v_) of 0.97%. The results demonstrate that VIS/NIR coupled with LRC-SR method can be a suitable strategy for the quick assessment of juiciness for pears.

## 1. Introduction

Pear is one of the most important fruits in the global fresh produce market. In the past decade, the global production of pears has been steadily increasing (2.26 million tons in 2010 and 2.37 million tons in 2018). Currently, pears are classified manually or automatically according to their external quality attributes. However, the visual attributes can only affect the initial purchase, while repeated sales are based on eating quality of the pear [1]. Therefore, the control of the internal quality of pear greatly affects sales and the profit space of the fruit industry [2].

Previous studies have determined that the juiciness is the indispensable quality attribute of pear [3,4,5,6]. Jaeger et al. [7] evaluated 10 samples from 6 genotypes through a consumer survey that most consumers describe the “ideal” pear as “sweet and juicy”, and Turner et al. [8] obtained a similar conclusion that the most important quality factors of pear recognized by consumers were texture, sourness and juiciness. Blanckenberg et al. [9] investigate the effect of harvest maturity on consumer preference for eating quality of European pears. About 67% consumers preferred pears high in melt character, juiciness and sweet taste. Therefore, juiciness is an important internal quality attribute of pear. Juiciness is a discriminant variable for evaluating pear quality, which represents the amount of liquid released on mastication [10,11]. During storage, juice production rate is the most variable sensory property, so it can help judge the freshness of fruit [12,13,14,15,16,17]. In addition, the juiciness can also influence people’s judgment on sweetness and sourness. Cano-Salazar et al. [18] proposed that juicer fruits release more sugars in the mouth than less juicy fruits do. Harker et al. [19] hypothesized that the texture of the fruit and the way it releases juice during chewing would influence the perception of sweetness, and Pasquariello et al. [20] proposed that differences in sweet taste were influenced by the breakdown of pear flesh during mastication rather than any inherent differences in the sugars and acids that exist in the fruit. Hence, juiciness is the significant quality attribute for the pear.

Currently, juiciness analysis is mainly performed with a trained panel according to ISO 11036:1994 standard [21]. This is an empirical method, but it has disadvantages, namely, it is time consuming, destructive and high priced. In addition, the empirical method relies on the tester’s judgment, and, thus, different people may get different results [22]. Furthermore, some scholars have proposed a method for measuring the juiciness using texture profile analysis (TPA) [23]. The TPA method mimics the occlusion process of human teeth, and the juiciness can be calculated by measuring the weight loss of the sample after the compression test [7,24]. Although the TPA method successfully quantified the juiciness by the machine, it is also a destructive analysis method, and the pear cannot be sold after measurement, thus, it cannot be used for pre-sale grading. Therefore, the development of a non-destructive method for detecting the juiciness of pear is a useful goal.

Visible−near-infrared (VIS/NIR) spectroscopy is an optical detection method, which has the advantages of being fast, non-destructive and convenient. In previous studies, many scholars predicted the internal quality of pears based on VIS/NIR spectroscopy [25]. Fan et al. [26] established prediction models (400–1000 nm) of soluble solids content(SSC) and firmness with the root mean square error of 0.491 and 0.721, respectively. Li et al. [27] applied partial least squares regression to establish a prediction model for sugar content of pear based on the three different wavelength ranges, with a determination coefficient of 0.92 and a root mean square error of 0.2%. Although there are no publications focusing on the detection of the juiciness of pear using non-destructive VIS/NIR spectroscopy, the juiciness of some other fruits (e.g., apples and peaches) were analysis by the spectroscopic method. Arefi et al. [28] classified fresh and mealy apples, which were identified by stiffness and juiciness based on biospeckle imaging. Mehinagic et al. [29] investigated the relationships between sensory attributes and non-destructive VIS/NIR spectroscopy. They indicated that there is a statistically significant relationship between VIS/NIR wavelengths and juiciness of apple, especially in the ranges of 670–710 and 920–950 nm. These results are in agreement with those of Watada et al. [30]. Baltazar et al. [31] evaluated the juiciness of more than 2000 fruits and established the prediction model based on VIS/NIR wavelengths in the ranges of 896–1684 nm. The coefficients of correlation were 0.86 and 0.83 for peaches and nectarines, respectively.

The physiological reasons for the juiciness changing in pear are mainly affected by three aspects, i.e., starch, pectin and moisture content. The first is the starch content in pear. At the initial stage of storage, the starch exists in a granular state, the texture of the pear is relatively solid, and the juiciness is negligible. Subsequently, the starch content and amylase activity first rapidly decreased and then gradually decreased during whole storage period [32]. The firmness decreased, but the flesh juiciness content increased as the starch changed. The second influencing factor is the changes in pectin. Pectin is a key substance involved in the mechanical strength of the primary cell wall, which is important to the physical structure of the fruit [33]. The flesh becomes elastic and juicy as the protopectin is hydrolyzed by the enzyme into soluble pectin. In addition, under the further action of enzymes, the fruit was finally softened and overripe, as the pectin was completely decomposed into galacturonic acid [33,34]. The third influencing factor is moisture content, which is gradually lost due to the presence of transpiration during storage. Therefore, the juiciness content is mainly affected by the hydrogen-containing compound, i.e., starch, pectin and the moisture content, which provides theoretical possibility for the non-destructive determination of the juiciness using VIS/NIR spectroscopy.

In summary, development of a non-destructive method for the detection of the juiciness of pear is necessary. Therefore, the objectives of this paper were to (1) evaluate the juiciness of pear using the VIS/NIR spectroscopy; (2) compare different spectral preprocessing methods for eliminating the light-scattering effects in the raw spectra; (3) utilize the competitive adaptive reweighted sampling (CARS) method to select the feature variables and enhance the model prediction ability; (4) evaluate the performance of the partial least squares (PLS) model based on the independent verification datasets.

## 2. Materials and Methods

### 2.1. Sample Collection

The experiment was carried out in the summer of 2019 in a fruit orchard at Qixia, Yantai, China (37° N, 120° E). A total of 127 crystal pears, a representative Asian pear, were selected for this research. The pear samples were sent back to laboratory immediately after picking. Samples were placed in a fresh-keeping box with ice packs during transport to avoid moisture loss. The experiment began on the next day after pear picking. Thirteen samples were randomly taken from the pears every 2 days for spectral scanning and juiciness measurement; the rest were kept at room temperature (25 °C) to obtain a gradient of storage time. The experiment lasted for 20 days.

### 2.2. Spectra Acquisition

**Figure 1:**
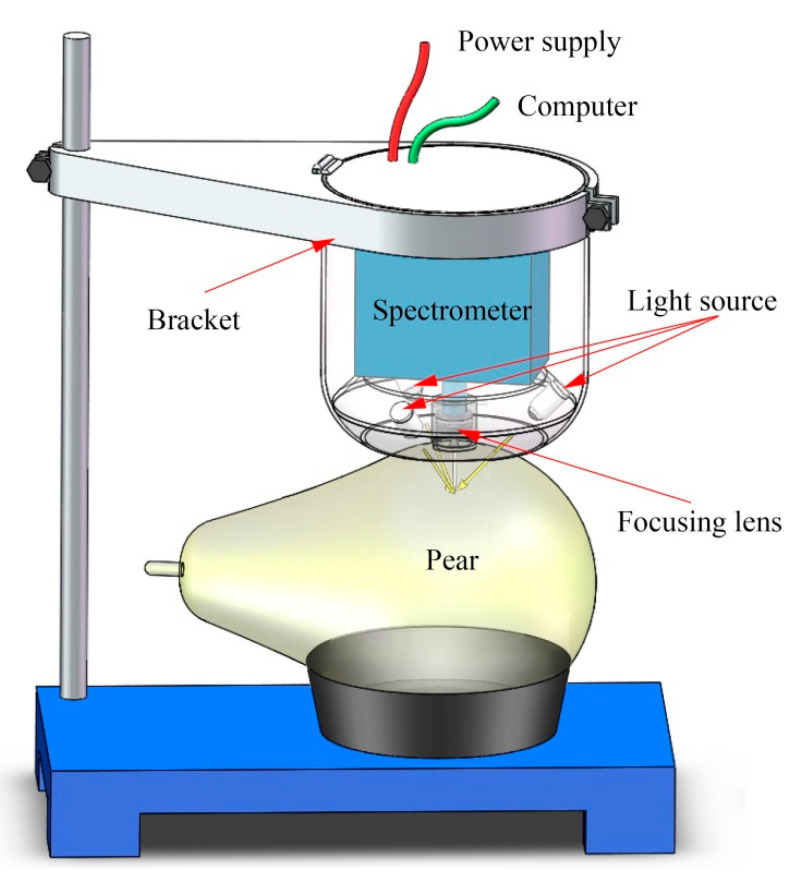
The visible−near-infrared (VIS/NIR) system for spectral measurement.

A VIS/NIR system was established for spectral measurement, as shown in Figure 1. The system mainly consisted of the light source, the spectrometer and the lifting bracket. The illumination unit consisting of three 5-watt halogen lamps (998070-1, Welch Allyn, Skaneateles Falls, NY, USA) and was adjusted at an angle of approximately 45° to prove a unit lighting. The spectrometer is the core part of the detection system. In this study, a VIS/NIR spectrometer (STS-NIR, Ocean Optics, Largo, FL, USA) with a range of 650–1100 nm and a resolution of 6 nm was used. A coated quartz lens (74-UV, Ocean Optics, Largo, FL, USA) was coupled to the front of the spectrometer. The diameter of the focusing lens is 5 mm and the focal length is 10 mm, which can converge the light to the entrance of the spectrometer to achieve a larger photosensitive range. A self-designed lifting bracket was used to fix the spectrometer and light source, providing the advantage of avoiding ambient light interference. The optical sensing system can achieve the acquisition of visible−near-infrared spectra from 650 to 1100 nm. The spectrum of this region has a deeper sample penetration and recorded many hydrogen-containing compound absorption bands, such as the starch, pectin and the moisture content [35,36]. When collecting the spectra of pears, we first put the pear on the sample tray, and then adjusted the height of the bracket to make the focusing lens close to the surface of the sample. We then set the integration time to 0.01 s and the average number to 5 times. The above operation was repeated three times accompanied by rotating the sample 120 degrees. The spectrum was expressed as transmittance (*T*%) using Equation (1).

(1) $$T=\frac{\left(I-D\right)}{\left(R-D\right)}\times 100\%$$

where T represents the transmittance of the sample; *I* is the interactance spectrum data of the sample; *D* is the spectrum data for dark current of the spectrometer without any illumination; *R* is the spectrum data reflected from a white teflon tile. In addition, each sample spectrum was repeatedly measured three times for averaging.

### 2.3. Reference Measurements of Juiciness

As a reference, the juiciness was defined as the ability to release juice on mastication of the pulp. After the spectra measured, the pears were peeled and the flesh of the spectral detection points were cut out using a circular sampling knife with a diameter of 0.015 m and a thickness of 0.01 m. A texture analyzer (TA.XTPlus, Stable Micro Systems, Inc., Surrey, UK) was used to obtain the juiciness. The samples were compressed twice to 70% deformation by a cylindrical plate with a diameter of 0.1 m at a speed of 8.3 × 10^−4^ m s^−1^. The juiciness of pears can be calculated by Equation (2) [37]:

(2) $$J=\frac{\left(W_{2}-W_{1}\right)}{W_{1}}\times 100\%$$

where J represent the juiciness of the pear sample; $W_{1}$ is the original weight of the pulp; $W_{2}$ is the weight after compression.

### 2.4. Chemometrics and Data Analysis

The raw VIS/NIR spectra not only contain the chemical composition information of the sample, but also reflect the light-scattering effects due to the different physical characteristics, such as the particle size of the sample [38]. The common multivariate linear regression methods such as the partial least squares (PLS), multiple linear regression (MLR) and principal component analysis (PCA) cannot effectively calibration of the acceptable results. Therefore, eliminating the scattering effects, including the additive effect and multiplicative effect, caused by changes in the physical properties of the sample is the key to accurate quantitative analysis of complex heterogeneous hybrid systems [38]. Different preprocessing methods, including normalization (NOR), first derivative (FD), detrend (DET), standard normal variate (SNV), multiplicative scatter correction (MSC), probabilistic quotient normalization (PQN), modified optical path length estimation and correction (OPLECm), linear regression correction combined with spectral ratio (LRC-SR) and orthogonal spatial projection combined with spectral ratio (OPS-SR) were used to eliminate the additive effect and multiplicative effect in the raw spectrum [38,39,40,41]. In addition to the above pretreatment methods, Savitzky−Golay smoothing, with second-order polynomial and 9 points of the window width, was used to reduce spectral noise before using the above preprocessing methods. The NOR, SNV, MSC, PQN, LRC-SR preprocessing methods were based on the assumption that the additive effect was not related to the wavelength and the raw spectrum could be express by the following equation (Equation (3)):

(3) $$X_{i}=a_{i}X_{i,\mathrm{chem}}+b_{i}l$$

where $X_{i}$ is the real raw spectrum of the *i*th sample; $a_{i}$ and $b_{i}$ are the additive effect and multiplicative effect, respectively; $X_{i,\mathrm{chem}}$ is the ideal spectrum that has the linear relationship with the chemical composition content; l is the unit vector. In addition, OPLECm and OPS-SR were based on the assumption that the additive effect was related to the wavelength, i.e., ($1,\;\lambda ,{\;\lambda }^{2}$), and the raw spectrum was express by the following equation (Equation (4)):

(4) $$X_{i}=a_{i}X_{i,\mathrm{chem}}+b_{i}l+c_{i}\lambda +d_{i}\lambda^{2}\;$$

where the coefficients $c_{i}$ and $d_{i}$ represent the additive effects that are related to λ and $\lambda^{2}$, respectively. λ represents the wavelength in the region of 650–1100 nm.

Effective wavelength selection can promote prediction accuracy and simplification of the PLS model [42,43,44]. Competitive adaptive reweighted sampling (CARS) was used in this study to evaluate the effectiveness of wavelength based on the different preprocessing spectra [45]. The CARS method used the adaptive reweighted sampling as the random selection method and the exponentially decreasing function as the performance of wavelength selection. After a plurality of random samplings, the wavelength points with a high absolute value of the regression coefficient were retained.

**Figure 2:**
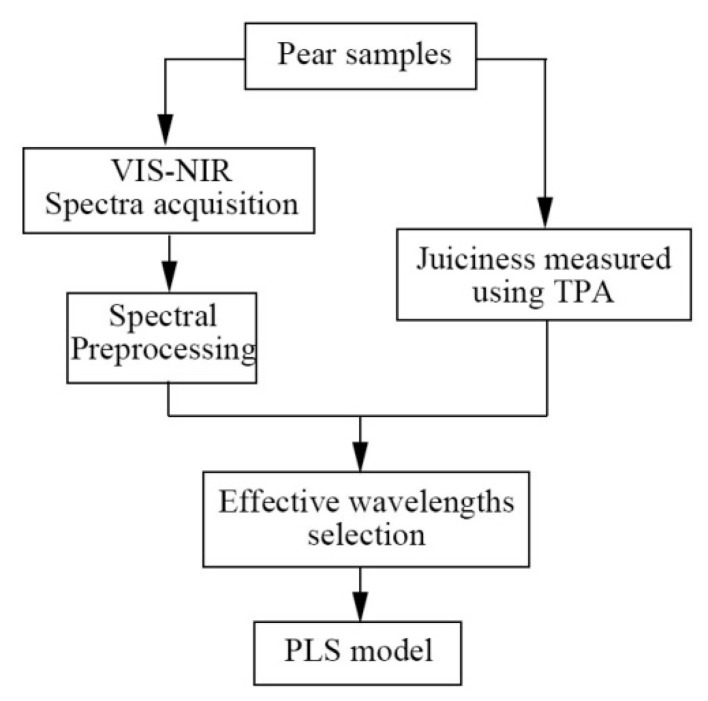
The flow chart of the analysis process.

PLS regression was used to perform statistic correlation of the VIS/NIR spectroscopy with the juiciness of pears [46,47]. Monte Carlo cross-validation (MCCV) was employed to assess the predictive power and determined the optimal number of latent variables (*LV*_s_) of the different models. Compared with other cross-validation methods, MCCV used random sampling to increase the credibility of model evaluation and reduce the risk of model over-fitting. Ten samples were randomly selected per experiment as calibration samples (a total of 100 pears) to establish the PLS model based on the cross-validation method, and the remaining samples were used for external verification (a total of 27 pears). The process was repeated 100 times, and the performances of the models were evaluated using the determination coefficient of cross-validation (*R*^2^_cv_) and root mean square error of cross-validation (*RMSE*_cv_).

(5) $$R_{\mathrm{cv}}^{2}=1-\frac{\sum_{i-1}^{n}{\left({\hat{y}}_{i}-y_{i}\right)}^{2}}{\sum_{i-1}^{n}{\left({\hat{y}}_{i}-y_{m}\right)}^{2}}$$

(6) $$RMSE_{\mathrm{cv}}=\sqrt{\frac{1}{N\times n}\sum_{i-1}^{n}{\left({\hat{y}}_{i}-y_{i}\right)}^{2}}$$

where ${\hat{y}}_{i}$ is the predicted value of the *i*th sample; $y_{i}$ is the measured value of the *i*th sample; $y_{m}$ is the average value of sample set; N is the times of repetition which is equal to 100 in this paper; *n* is the total number of samples. The flow chart of the analysis is shown in Figure 2.

## 3. Results

### 3.1. Juiciness Parameter Distributions

**Figure 3:**
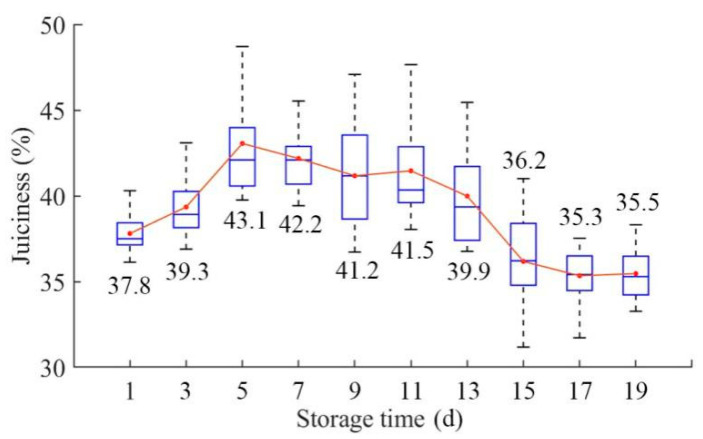
Effects of storage time on the juiciness of pears.

The juiciness distributions of pears during the whole storage period are shown in Figure 3. With an increase in the storage time, the average juiciness tended to rise first and then decrease. The average juiciness of pears reached its highest value on the fifth day. After the fifth day, the fruit began to decay and the intercellular fluid was lost, resulting in a decrease in juiciness and flavor. This change was similar to the research conclusion of Hohn et al. [48]. This trend may be determined by the combined effect of the increase in soluble substances caused by the degradation of starch and pectin in the early storage period and the continuous decrease in moisture content caused by the volatilization of water [32,33,34]. In addition, the changing regularity of juiciness may vary in different storage environments [49].

**Figure 4:**
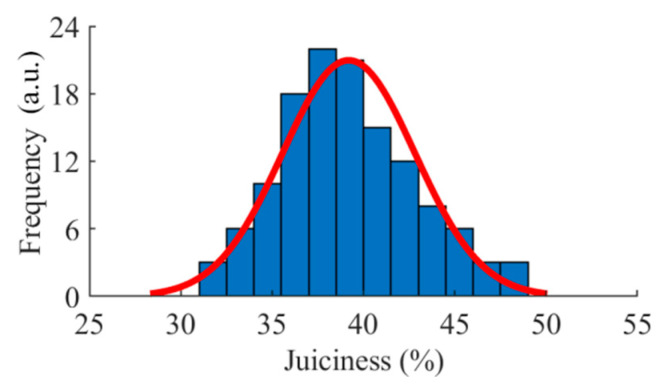
The juiciness distribution of all pears during storage. a.u. stands for arbitrary unit.

The juiciness content of 127 pears covered 31.18 to 48.71%. As shown in Figure 4, the concentration of the sample was approximately in accordance with the normal distribution. Ten samples were randomly selected every day as modeling samples (a total of 100 pears), and the remaining were used for external verification (a total of 27 pears). The juiciness distribution of the calibration set and the external verification set are shown in Table 1.

### 3.2. Spectra and Spectra Analysis

**Figure 5:**
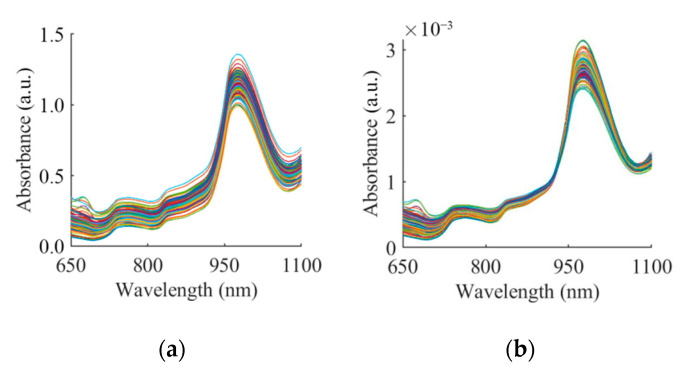

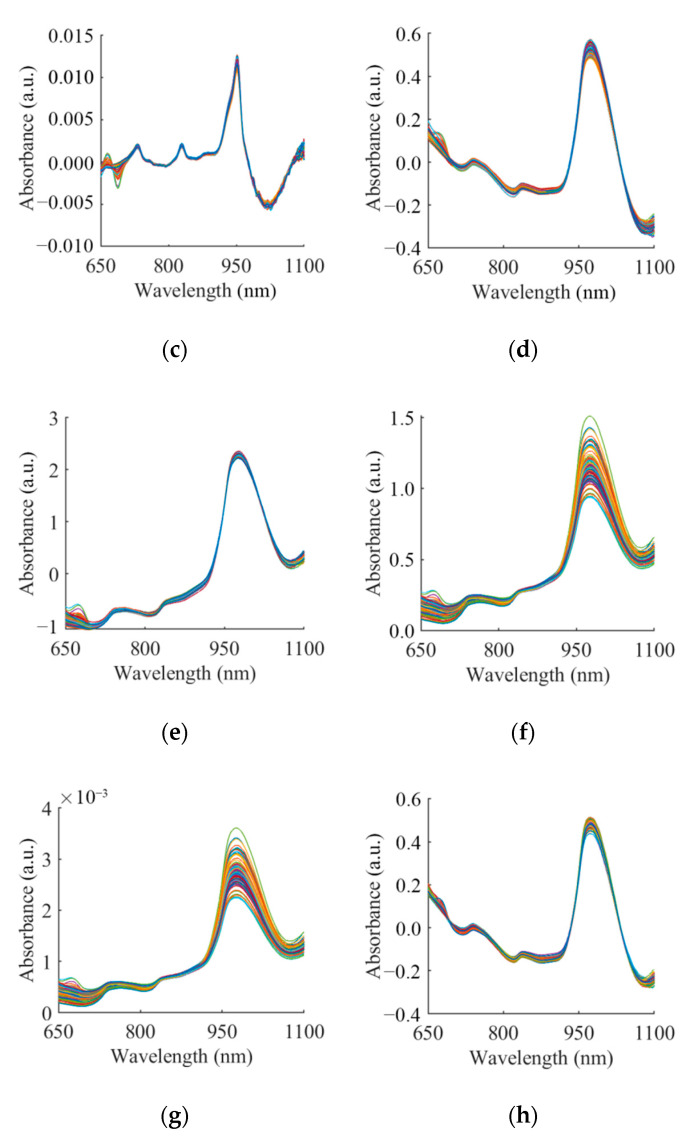
(**a**)The raw absorbance spectra (RAW) and the spectra after (**b**) normalization (NOR), (**c**) first derivative (FD), (**d**) deterred (DET), (**e**) standard normal variate (SNV), (**f**) multiplicative scatter correction (MSC), (**g**) probabilistic quotient normalization (PQN), and (**h**) modified optical path length estimation and correction (OPLECm) pretreatment methods. a.u. stands for arbitrary unit.

Typical absorbance spectra for the pears showed a number of clear absorption peaks (Figure 5a). For different storage periods, similar peaks can be observed at the wavelengths of 675, 740, 840 and 975 nm. From 650 to 700 nm, there was a decrease in absorbance with a small peak at about 675 nm, which was related to the absorption of chlorophyll in pears [50,51]. The absorbance of the peak here was determined by the content of chlorophyll in the sample. After the trough at 700 nm, there was a continuous increase in absorbance; the second and the third peak appears around 740 and 840 nm, respectively. The peaks here were related to the absorption of water and carbohydrate. Due to the combined effects of -OH (940 nm) and water (980 nm), the strongest absorption band exists at ~975 nm [52]. In general, a considerable baseline offset was observed in the 650–1100 nm region. In addition, different preprocessing spectra showed similar results in which the same absorption peaks were observed, as shown in Figure 5.

The significant additive and multiplicative effects could be seen from the raw spectra. Furthermore, the scattering effects, including additive and multiplicative effects, could be eliminated after the preprocessing methods. As shown in Figure 5, the characteristics of the spectrum were more obvious after preprocessing based on the five different methods. In addition, OPS and LRC reduced the addition effect based on the two different assumptions described in Section 2.4. After the additive effect had been eliminated, the multiplication effect could be corrected by a simple but effective calculation, i.e., the spectral ratio method [38]. In theory, the elimination of the light-scattering effects would improve the effectiveness of spectral variables, which was evaluated by the correlation coefficient in this paper.

**Figure 6:**
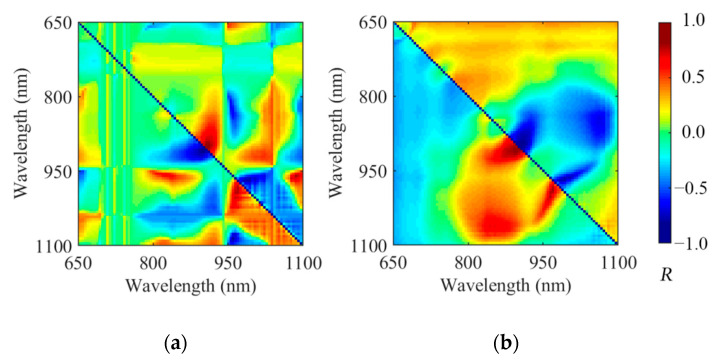
The spectral correlation after (**a**) orthogonal spatial projection combined with spectral ratio (OPS-SR) and (**b**) linear regression correction combined with spectral ratio (LRC-SR).

The single-variable correlations between the spectra data points and juiciness content were compared and analyzed. The correlation between the original spectrum and the juiciness was between 0.1 and 0.4. The additive and multiplicative effects of the spectra were reduced after different pretreatments and some wavelengths related to chemical bond absorption were highlighted; notably, for the SNV preprocessing, the maximum of the correlation coefficient was equal to 0.7 at 1015 nm. The spectral correlation after OPS-SR and LRC-SR processing was shown in Figure 6, which was much better than the former seven preprocessing methods. The abscissa and ordinate represented the molecular and the denominator wavelength of the spectrum ratio; the color of each pixel in the image represented the correlation between the spectrum ratio and the juiciness. After the simple spectral ratio calculation, the correlation between the variables and the juiciness was significantly improved. The highest univariate correlations of OPS-SR and LRC-SR were 0.79 and 0.83, which were calculated by $\frac{X_{\lambda =895}}{X_{\lambda 9005}}$ and $\frac{X_{\lambda =890}}{X_{\lambda =900}}$, respectively (**X**_λ_ represents the spectral value at wavelength of λ). In addition, the ratio variable of spectra improved the correlation between the spectral information and chemical composition content, but the redundancy data were also greatly increased. Therefore, in order to improve the accuracy and reduce the complexity of the model, the wavelength validity of the preprocessed spectrum was evaluated by the CARS method.

### 3.3. PLSR Models Based on the Characteristic Wavelength

**Figure 7:**
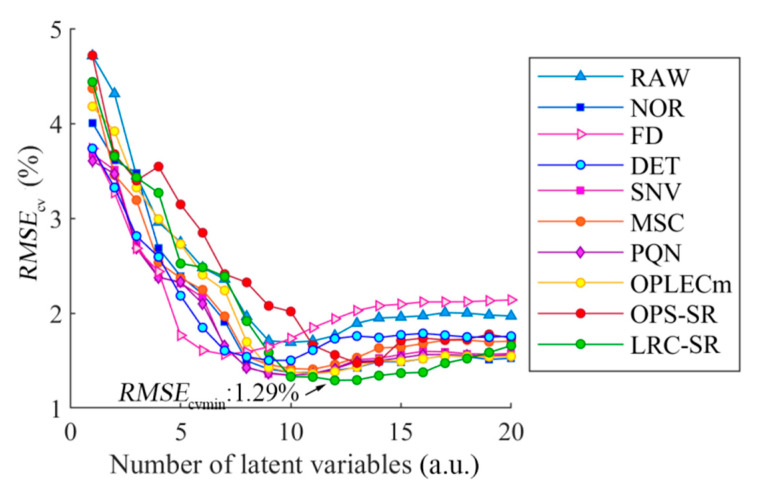
Root mean square errors of partial least squares regression models for the cross-validation of juiciness with 1–20 latent variables. *RMSE*_cv_ represents the root mean square error of cross-validation, *RMSE*_cvmin_ represents the minimum value of *RMSE*_cv_, a.u. stands for arbitrary unit.

The prediction model of juiciness in pear samples was established by the PLS method based on the different preprocessing spectrum, and the root mean square error of Monte Carlo cross-validation was used to determine the number of number of latent variables, as shown in Figure 7.

The value of *RMSE*_cv_ was significantly reduced when modeled by the preprocessing spectral datasets. For the determination of the *LV*_s_, the raw spectra and preprocessing spectra namely, DET, SNV and MSC, selected 10 *LV*_s_, and the *RMSE*_cv_ values were 1.69%, 1.51%, 1.34% and 1.35%, respectively. NOR, PQN and OPLECm selected 11 *LV*_s_, and the *RMSE*_cv_ was 1.36%, 141% and 1.36%, respectively. The OPS-SR and LRC-SR models were the most complex, with 13 and 12 *LV*_s_ selected; the *RMSE*_cv_ was 1.47% and 1.29%, respectively. FD chose the least number of *LV*_s_; however, the effect of FD was not ideal, which can be explained as the noise in the spectrum was amplified after the derivative processing. When comparing all of the spectral data modeling results, including the raw spectra and nine different preprocessing spectra, the prediction performance of LRC-SR was the optimal result. In addition, the CARS method was processed according to the optimal *LV*_s_ selected by MCCV. Furthermore, the raw spectra and the spectra after FD, DET, NOR, SNV, MSC, PQN, OPLECm, OPS-SR and LRC-SR retained 14, 35, 21, 23, 28, 20, 54, 48, 48 and 19 wavelengths, respectively. The *R*^2^_cv_ and *RMSE*_cv_ modeling by the characteristic wavelengths based on the different spectra are shown in Table 2.

The accuracy of the model based on characteristic wavelengths was significantly better than the result of all wavelengths. The biggest improvements were LRC-SR preprocessing datasets, and the PLS model reduced the error by approximately 20% under the condition of using 10% variable information. This showed that the LRC-SR preprocessing method should be combined with variable selection steps to obtain a more accurate and simpler model. In addition, the modeling result was similar to that of Li et al. [38]. In this research, LRC-SR preprocessing datasets finally retained 19 variables (26 wavelengths), including 668, 677, 687, 696, 701, 733, 738, 752, 757, 762, 766, 776, 818, 828, 851, 861, 871, 880, 890, 900, 919, 1006, 1011, 1060, 1065 and 1075 nm. When comparing OPS-SR and LRC-SR, the additive effects of pear could be wavelength independent in a similar way to those of apple [38], and the multiplicative effect in the raw spectra could be eliminated by the simple spectral ratio method.

### 3.4. External Verification of the Model

The verification sets, including 27 samples, were used to further verify the prediction performance of the PLS models established by the characteristic wavelengths based on the different preprocessing datasets. The spectra and the reference juiciness content of these pears in the verification sets were collected as described in Section 2.1. The partial least squares regression modeling results of the external verification for pear juiciness based on the characteristic wavelengths using different preprocessing spectra are shown in Table 3.

**Figure 8:**
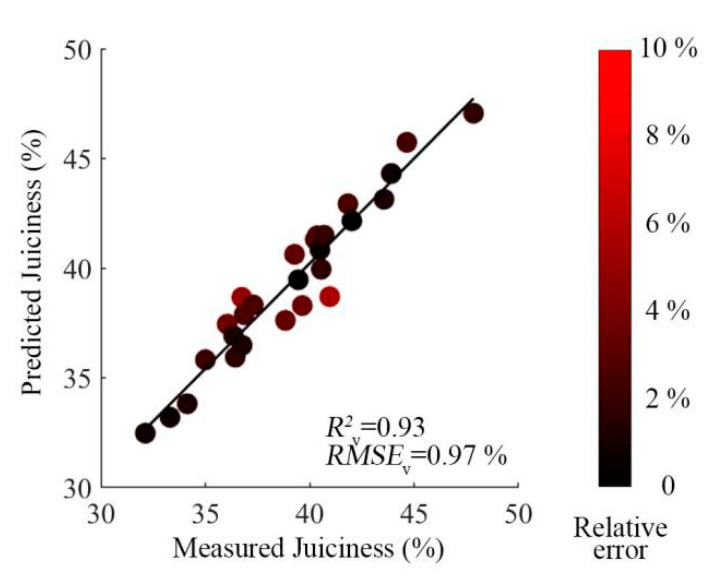
Scatter plots of the simplified models for the prediction after LRC-SR preprocessing methods.

LRC-SR still showed the best prediction accuracy: the determination coefficient of external verification (*R*^2^_v_) was 0.93, and the root mean square error of external verification (*RMSE*_v_) was 0.97%. The results of OPS-SR were also acceptable, but from the scatter plot, the result shows that the linearity of the model and the prediction errors for juiciness content were lower and larger than the prediction value of the LRC-SR method, respectively. In general, the accuracy of the external verification of the prediction set was encouraging. However, the performance of other preprocessing methods, such as that of the NOR and PQN method, was not satisfactory due to the failure in elimination of the light-scattering effects in the raw spectra. The scatter plots of the juiciness predicted by LRC-SR combined with the CARS method and measured juiciness are shown in Figure 8, and the relative deviation of each sample was color-coded.

## 4. Discussion

In this work, nine pretreatment methods, namely, NOR, FD, DET, SNV, MSC, PQN, OPLECm, OPS-SR and LRC-SR, were compared [38,39,40,41]. Among these methods, FD and DET eliminated the additive effect. The difference was that DET considered that the additive effect was different as the wavelength changed. Other preprocessing methods considered both the additive effect and the multiplication effect. NOR, SNV, MSC, and PQN are traditional methods, which treat the multiplication effect as a fixed value for different wavelengths, while OPLECm, OPS-SR and LRC-SR are the latest methods which take into account the fact that the multiplication effect changes with wavelength [38]. The additive and multiplicative effects of the spectra were reduced after pre-processing, and the correlation between the spectral datapoint and the juiciness content was significantly improved. When comparing the nine preprocessing methods, LRC-SR combined with the CARS method obtained the optimal prediction effect: the determination coefficient of external verification was 0.93, and the root mean square error of external verification was 0.97%, respectively. The LRC-SR was composed of two steps: first, the additive effect could be estimated and eliminated by the linear regression correction, and, second, the multiplicative effect was eliminated by a simple spectral ratio calculation. Compared with other preprocessing methods, LRC-SR possessed the advantages of high accuracy and simple calculation and, thus, was most suitable for use in the practice of prediction of the juiciness of pear samples [38].

However, we noticed that the prediction model based on all wavelengths processed by OPS-SR and LRC-SR required too many latent variables. This was because although the ratio algorithm can improve the linear correlation between the spectra data points and chemical composition content, it also greatly increases the complexity of the data. Therefore, the CARS method, as an indispensable step for LRC-SR preprocessing, was used to select the effective variables to further improve the PLS model prediction ability and simplify the calculation. With the processing of CARS, the raw spectra and the spectra after NOR, FD, DET, SNV, MSC, PQN, OPLECm, OPS-SR and LRC-SR retained 21, 23, 14, 35, 28, 20, 54, 48, 48 and 19 wavelengths, respectively. Furthermore, the latent variables of all prediction models were declined. The juiciness prediction model with the lowest errors was established by SNV and LRC-SR combined with CARS after pretreatment. LRC-SR preprocessing datasets retained 19 wavelength variables (26 wavelengths), including 668, 677, 687, 696, 701, 733 nm, 738, 752, 757, 762, 766, 776, 818, 828, 851, 861, 871, 880, 890, 900, 919, 1006, 1011, 1060, 1065 and 1075 nm. The emphasis of these characteristic wavelengths provides the possibility to explain the model from the perspective of chemical bond absorption.

According to fruit physiology, the release of juice is mainly affected by starch, pectin and moisture content. Pasquariello et al. [20] suggested that weight (water) loss during cold storage is positively correlated with juiciness, and that the correlation coefficient is equal to −0.48. The obtained effectiveness variables by LRC-SR preprocessing combined with the CARS method for prediction of juiciness included several water-related wavelengths, such as 752, 757, and 980 nm (the third overtone of -OH) [52]. However, the juiciness was notably different from the water in the fruit, which has been confirmed by many scholars. Baltazar et al. [31] observed that the juiciness of peach is related to the availability and state of the water in the fruits rather than water content. Iwanam et al. [12] indicated that the juiciness of apples is the combination effect of water content, firmness, and apoplastic solution. Kappel et al. [3] and Chen et al. [15] also believe that the juiciness exists in a linear relationship with the firmness of the fruit. Among the effectiveness wavelengths we reserved, 668, 678, 687, 696, 738, 890, 980 and 1060 nm may contain information related to firmness, which was also reported by Fan et al. [26] and Yu et al. [53]. Furthermore, Vangdal [54] gave a clearer explanation for the relationship between juiciness and firmness, and they believed that the juiciness is indirectly related with physiology, cell structure and starch hydrolyses in fruit. Additionally, we noticed that the variables around 1060 nm were summarized as the absorption band related to starch, which has also been reported by other researchers [55]. Therefore, the selection of wavelengths may represent the absorption of starch, pectin and moisture content. In addition, the changing of the starch and pectin content also affects the value of the firmness in fruit. The juice released by pear samples contained soluble sugar and acid, and, thus, the feather variables at 701, 733, 762, 766, 776, 828, 919, and 1011 nm were also selected as the modeling bases in this research. In Yu and Li’s research [53,56], these wavelengths were also considered to be the soluble solids’ absorption band. The selection results could also explain that the juiciness can affect people’s perception of sweetness [20]. The released juice carried soluble sugars that can be tasted directly, and, thus, the juiciness should be considered one of the most important evaluation parameters for the taste of fruit.

The sample used in this experiment is Crystal Pear, a typical thin-skinned Asian pear with a diameter of about 65 mm. Three 5-watt halogen lamps were installed together to produce a 15mm diameter spot, as shown in Figure 1. The spectrometer-coupling lens was located in the center of the spot and close to the surface of the sample. This detection scheme has a small contact area with a sample surface to ensure spectral stability and prediction accuracy when the sample diameter changes in a small range. However, when we use the juiciness calibration model of the Crystal Pear to predict other varieties of pears, such as *Pyrus sinkiangensis* and Hosui pears, the accuracy of the model may be reduced due to it being affected by skin and color [51,57]. Vaudelle et al. [58] also indicated that the changes in size and peel have a great influence on the optical parameters of apples. In further research, a cost-effective portable device for the inspection of internal quality attributes of pear should be developed by using the important wavelengths and proposed methods. Furthermore, other varieties of pear should be included in the modeling to obtain a more stable and applicable juiciness prediction model.

## 5. Conclusions

This research provides a non-destructive method to detect the juiciness of pear. In a pretreatment comparison study, it was found that the combination of linear regression correction (LRC) and spectral ratio (SR) processing was better than the other pretreatment methods. In addition, the competitive adaptive reweighted sampling (CARS) can optimize the characteristic wavelength of the spectrum and effectively improve the accuracy and stability of the model. The partial least squares regression model (PLS) based on characteristic wavelengths was externally validated using 27 samples. For the external verification, the determination coefficient of juiciness was 0.93, and the root mean square error was 0.97%. The result demonstrated that the VIS/NIR spectrometer was a reliable tool to monitor the juiciness of pears during storage. The results provide a more comprehensive reference for fruit picking and storage.

## Figures and Tables

**Table 1:** Juiciness statistics of the calibration set and external verification set.

Sets	Number of the Samples	Juiciness (%)		
		Maximum	Minimum	Average
**Calibration set**	100	48.5	31.2	39.2
**External verification set**	27	47.9	32.2	39.1

**Table 2:** The partial least squares regression modeling results of the cross-validation for pear juiciness based on the characteristic wavelengths using different preprocessing spectra.

Preprocessing Methods	Latent Variables	Number of the Wavelength Variables	*R* ^2^_cv_	*RMSE*_cv_ (%)
RAW	9	21	0.88	1.24
NOR	9	23	0.91	1.18
FD	5	14	0.86	1.37
DET	10	35	0.89	1.20
SNV	9	28	0.91	1.02
MSC	8	54	0.91	1.06
PQN	9	20	0.90	1.09
OPLECm	10	48	0.92	1.03
OPS-SR	10	48	0.91	1.06
LRC-SR	8	19	0.93	0.94

**Table 3:** The partial least squares regression modeling results of the external verification for pear juiciness based on the characteristic wavelengths using different preprocessing spectra. *R*^2^_v_ represents the determination coefficient of external validation, *RMSE*_v_ represents the root mean square error of external verification, a.u. stands for arbitrary unit.

Preprocessing Methods	*R* ^2^_v_	*RMSE*_v_ (%)	Max Relative Error (%)	Mean Relative Error (%)
RAW	0.88	1.30	7.5	2.7
NOR	0.83	1.53	6.7	3.3
FD	0.86	1.49	8.0	3.2
DET	0.87	1.30	7.9	2.8
SNV	0.88	1.28	8.1	1.8
MSC	0.88	1.26	8.1	2.7
PQN	0.86	1.48	8.1	3.2
OPLECm	0.87	1.33	7.8	2.8
OPS-SR	0.91	1.06	5.6	2.3
LRC-SR	0.93	0.97	5.7	2
